# Evaluating Factors Impacting Polymer Flooding in Hydrocarbon Reservoirs: Laboratory and Field-Scale Applications

**DOI:** 10.3390/polym16010075

**Published:** 2023-12-26

**Authors:** Abdelaziz L. Khlaifat, Sherif Fakher, Gbubemi H. Harrison

**Affiliations:** 1 Petroleum and Energy Engineering, School of Sciences and Engineering, American University in Cairo, New Cairo 11835, Egypt; sherif.fakher@aucegypt.edu; 2 Chemical and Petroleum Engineering Department, American University of Ras Al Khaimah, Ras Al Khaimah 72603, United Arab Emirates; gbubemi.harrison@aurak.ac.ae

**Keywords:** polymer injection, polymer types, mobility and resistance factor, field applications, tertiary oil recovery

## Abstract

Polymer flooding is an enhanced oil recovery (EOR) method used to increase oil recovery from oil reservoirs beyond primary and secondary recovery. Although it is one of the most well-established methods of EOR, there are still continuous new developments and evaluations for this method. This is mainly attributed to the diverse polymers used, expansion of this method in terms of application, and the increase in knowledge pertaining to the topic due to the increase in laboratory testing and field applications. In this research, we perform a review of the factors impacting polymer flooding in both laboratory studies and field-based applications in order to create guidelines with respect to the parameters that should be included when designing a polymer flooding study or application. The main mechanism of polymer flooding is initially discussed, along with the types of polymers that can be used in polymer flooding. We then discuss the most prominent parameters that should be included when designing a polymer flooding project and, based on previous laboratory studies and field projects, discuss how these parameters impact the polymer itself and the flooding process. This research can provide guidelines for researchers and engineers for future polymer flooding research or field applications.

## 1. Introduction

Polymer flooding is the most commonly applied chemical enhanced oil recovery method (CEOR) globally. This is primarily due to its low cost compared to other CEOR methods such as surfactant flooding. Also, because it is a well-established technique, its application is well known amongst reservoir engineers globally [1,2,3,4,5]. Although this technique is well known, many polymer flooding applications still fail in reservoirs. This is due to the complexity of reservoirs and the attempt to expand the application of polymer in new fields and applications, such as microfluidics [6,7,8]. Due to the vast scale of polymer flooding and types of polymers [9,10,11], it is important to provide an updated guide to the factors that could impact the application of polymer flooding in both laboratory studies and field projects.

The use of polymer injection to improve sweep efficiency by increasing the viscosity of the injected water was tested for the first time in 1944 by Detling [12]. The first experimental results supporting Detling’s findings were published by Pye [13] and Sandiford [14]. The first ever field applications of polymer flooding were reported in the USA in the late 1950s [13,14]. Afterwards, many lab experiments, as well as pilot and field-scale tests results for polymer flooding as an essential EOR method, appeared in the literature [15,16,17,18,19,20,21,22,23,24,25,26,27,28,29,30,31,32,33]. Over 300 field applications have been reported worldwide [30]. Reported incremental recovery in oil fields ranges from 6 to 27%, averaging 21% of the original oil in place. Water cuts were brought down to the 4–39% range, averaging 17% [31]. When polymer is added to the injected water, it increases its viscosity, making it move slower than oil in the reservoir, resulting in an increase in the overall sweep efficiency and, consequently, the recovery factor [11,12,13,14,15,16,17,18,19,20,21,22,23,24,25,26,27,28,29,30,31].

Many studies have investigated polymer flooding to improve oil recovery and reduce water production. Nadirov [34] simulated the reduction in friction losses associated with polymer injection through the addition of drag reduction agents. Li [35] modeled fluid–particle interactions using computational fluid dynamics and the discrete element method in order to predict polymer injectivity in porous media while taking into consideration fluid-induced fractures, water quality, and polymer rheology. Rashid [36] conducted a simulation study of a novel model using data from laboratory kinetics, rheology data, high resolution surveillance of the injector, and a comprehensive analysis of the produced fluid. Anand [37] evaluated the use of water-alternating polymer to enhance polymer pattern performance and optimize polymer utilization through reservoir simulation. Lu [38] used lab-measured parameters of a synthesized polymer to run a simulation in order to optimize polymer flooding in the Daqing field. Simulation results were used as a precursor to an actual field testing. Hwang [39] developed an injectivity model for polymer injection in horizontal wells to evaluate the factors impacting the success of polymer flooding in the wells. Leon [40] conducted a simulation study to evaluate the application of polymer flooding in a high-temperature and high-salinity carbonate reservoir. Gandomkar [41] performed a geomechanical simulation to evaluate the ability of a newly developed porous shape memory polymer to reduce the production of sand from unconsolidated and friable formations.

Therefore, in this research, we attempt to provide guidelines with respect to the factors impacting polymer flooding and explain each factor and how to overcome it for both laboratory studies and field applications of polymer flooding. The focus of this research is on different applications of polymer flooding in the laboratory, as well as the methods by which polymers can be degraded in the field. This can help to reduce polymer flooding problems and failures in both laboratory applications and field studies.

## 2. Polymer Flooding Principles and Mechanisms

Polymer flooding is considered a mobility control method. It can help to considerably improve areal and vertical sweep efficiencies if applied correctly; it can also help to increase the displacement efficiency. Mobility is defined as the ratio between a fluid’s permeability and its viscosity. As the mobility of a fluid increases, its ability to flow also increases. This corresponds to an increase in the fluid’s permeability and/or a decrease in its viscosity. Therefore, it is always preferable to increase the mobility of oil to increase the overall recovery [36].

When applying water flooding, as long as the mobility of the oil is higher than the mobility of the water, the oil flow behavior will be better; therefore, higher oil recovery will be observed. Since it is possible to calculate oil mobility, the same can be applied to water by dividing the water permeability by its viscosity. By comparing both the permeability of water and oil, it becomes clear which phase will flow more competitively. A common method to compare the two mobility values is the use of the mobility ratio. This is defined as the ratio between the mobility of the displacing phase and that of the displaced phase [33,34,35,36,37,38,39,40]. In all secondary and tertiary recovery applications, the displaced phase is the oil, whereas the displacing phase is the injected fluid that is being used to displace the oil. Based on this, it is always advisable to maintain a higher mobility of the displaced phase (oil) than that of the displacing phase (e.g., water or polymer). This is why the mobility ratio should be maintained at less than one for a favorable displacement process.

Water can suffer from a premature increase in mobility ratio for two main reasons. First, it has a low viscosity value, which is comparable to or sometimes even less than that of oil. This can result in the water having a lower resistance to flow compared to oil, which, in turn, creates channels through the oil zone—a phenomenon referred to as viscous fingering [18,19,20,21,22,23,24,25,26,27,28,29,30,31,32,33,34,35,36,37,38,39,40,41]. Secondly, depending on the water saturation and the wettability, the water can have a higher permeability than the oil. These two factors can result in a significant increase in the mobility ratio and, thus, a significant reduction in the overall oil recovery.

The proposed solution to the premature increase in the mobility of the water is to target the two underlying causes of this increase. This can be achieved by significantly increasing the viscosity of the displacing phase (water). This also reduces the permeability of the water relative to the oil and creates a more uniform flood front. Therefore, a chemical is added to the water to increase its viscosity significantly beyond that of the oil; this is the fundamental concept of polymer flooding. By varying the type of polymer and its concentration, extremely high-viscosity values can be achieved [18]. This significantly decreases the mobility of the displacing phase and, thus, reduces the value of the mobility ratio below one for a longer duration. Based on this concept, polymer flooding is primarily classified as a mobility control method, since it maintains the mobility ratio below unity for as long as possible [3].

## 3. Types of Polymers

Polymers are defined as long-chain chemicals composed of bonded, simply structured monomers. A polymer can be made of one monomer or multiple monomers, referred to as copolymers or a terpolymers [42,43,44]. The selection of the monomers depends on multiple factors, including:

- Temperature: The ability of the polymer to withstand high temperatures depends on an innate property of the polymer itself. This is a strong function of the main compounds that form the monomer; the type and orientation of the bonds present within the monomer structure; and, finally, the bond strength between the individual monomer units that form the long-chain polymer. The measure of the ability of the polymer to resist degradation under a specific temperature is referred to as thermal stability. Depending on the type of polymer, the temperature range for thermal stability can be extremely high according to previous research, ranging from 31 °C to 212 °C [45]. It is also important to note that for a specific polymer, multiple thermal stability ranges can be mentioned. The variation in the thermal stability values is the result of two main parameters. The first is the molecular weight of the polymer; this can vary significantly for the same polymer depending on the initiator used to link the monomers; the purity of the monomers; and the monomer density, which is reflected directly in the molecular weight, since the definition of molecular weight is the number of grams of the chemical per mole. The variation in thermal stability could also be due to variation in testing conditions [46]. Some researchers have focused on using freshwater and neutral pH when measuring thermal stability, whereas others have used tap water, which has a variety of salinity and pH values depending on the country and location. It is therefore highly advised to measure the thermal stability of each polymer individually before testing, since it may vary relative to the calculated value [47].
- Pressure: Pressure is usually divided into two main types: injection pressure and the pressure differential during propagation into the formation. The ability of the polymer to withstand high pressure without failing is important to evaluate to avoid premature or excessive polymer degradation. It can be measured using pressure differential laboratory experiments involving core flooding [44,45,46,47].
- pH: Different polymers can tolerate a wide range of pH values depending on the monomer used to develop the polymer. Although a wide range of polymers can function well under high pH values or basic conditions, a limited number of polymers can resist degradation under low pH values or acidic conditions. These polymers can be applied under highly acidic conditions such as in the presence of high concentrations of hydrogen sulfide or carbon dioxide or when the crude oil has a high total acid number. It is important to note that these types of polymers are usually much more costly than other conventional polymers [48,49,50].
- Salinity: When referring to salinity, there are two main categories of interest: monovalent cations and divalent cations. Monovalent cations include any salt that is formed of an element with one valence electron, such as sodium, while divalent cations are salts that are formed of an element with two valence electrons, such as calcium. Salts have an overall negative impact on polymers, with monovalent cations being less damaging compared to divalent cations. A detailed description of the impact of salt in field applications of polymer flooding is presented in [33].
- Rock Type: The type of rock involves both its lithology and its properties. The lithology of rock has an impact on the polymer if it can react with the polymer itself, causing polymer structural change and, eventually, degradation. The rock property that has the most significant impact on the polymer is wettability. If the polymer has a tendency to adhere to the rock surface, a large volume of the injected polymer is lost in the formation, failing to perform its function. This results in a significant increase in the polymer flooding cost [42,43,44,45].
- Polymer Molecular Weight: The specific molecular weight of the polymer depends on the selected type of polymer and the method of polymerization. The molecular weight of the polymer impacts the concentration of polymer added to the water prior to injection, the injectivity of the polymer, and the ability of the polymer to propagate in different pore sizes in the formation. It is therefore extremely important to measure the molecular weight of the polymer prior to injection. It is also important to note that the molecular weight of the polymer can change over time due to polymer degradation [42,43,44,45,46,47,48,49,50].
- Polymer Cost: The overall cost of a polymer flooding operation relies on multiple components, including the cost of the polymer itself. Depending on the polymer used, the cost can be extremely low, while for more specialized polymers, the cost of the polymer can be extremely high. The selection of the polymer itself is dependent on reservoir rock and fluid properties, reservoir thermodynamics, and operational considerations [49].

Polymers are usually characterized by several distinct properties. These properties govern the selection of the polymer and the ability to use that specific polymer under given conditions, as mentioned previously. The polymers used in EOR applications need to be able to withstand the harsh environments of the oil reservoirs. This includes high-temperature and high-pressure conditions, which could result in polymer shearing, dehydration, degradation, and syneresis. The polymer should also be able to resist high salinity, including monovalent and divalent cations such as sodium, calcium, and magnesium. Finally, the polymers must be able to perform well under acidic conditions due to the occasional presence of hydrogen sulfide and carbon dioxide. Although there are hundreds of types of polymers present, they can be grouped into two general categories: organic and inorganic polymers.

### 3.1. Organic Polymers

Examples of such polymers are xanthan gum (produced by the bacteria Xanthomonas campestris) and polysaccharides (obtained from sugar in a fermentation process caused by the bacterium Xanthomonas campestris). The molecular structure of polysaccharide biopolymers provides the molecules with considerable stiffness [51], so they behave like rigid-rod molecules [52]. Such a molecular structure and behavior allow the polysaccharide biopolymer to be salt-resistant; therefore, the viscosity of its solution is not affected by salinity. The main advantage of biopolymers compared to synthetic polymers is their environmental friendliness due to their organic nature. This type of polymer is not widely used to enhance oil recovery due to its high cost and its stability degradation at high temperatures (>200 °F).

HPAM types of polymers are much more widely used than biopolymers, because HPAM has good water solubility and good surface activity, as well as stable rheological properties; it also offers advantages in terms of price and large-scale production, and its solutions exhibit significantly greater viscoelasticity than xanthan solutions. Polymers are used in aqueous solutions at low concentrations of 300 ppm (0.03%) to less than 2000 ppm (0.20%).

### 3.2. Inorganic Polymers

**Figure 1:**
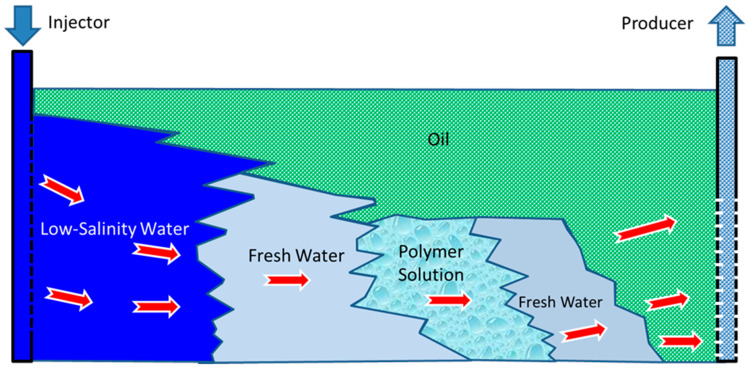
Polymer flood injection mechanism.

An example of such a polymer is partially hydrolyzed polyacrylamide (HPAM), which is obtained by the polymerization reaction of acrylamide monomer [53,54,55,56,57]. Dissolving the obtained long molecular chain of HPAM in fresh water makes the fluid flow through tortuous porous media with ease. When the salinity of the water increases, the electrolytes in the polymeric solution cause the molecules to form a spiral shape that impedes the flow through the porous space and reduces the viscosity of the solution. Due to its sensitivity to salts, HPAM is usually injected between two slugs of fresh water, as shown in Figure 1. HPAM solutions are vulnerable to the presence of oxygen, high temperatures, and mechanical degradation. At and near the wellbore, high fluid velocities and temperatures may result in the breaking down of the long chains of HPAM molecules. HPAM is widely used for EOR due to its low cost and high resistance to being driven out of the formation [54].

## 4. Polymer Flooding—Lab Scale

Many studies have investigated the use of polymers for different applications in laboratory-based experiments. These experiments investigated either the ability of a specific or a newly synthesized polymer to improve oil recovery or the impact of a specific parameter on polymer performance and degradation [34,35,36,37,38,39,40,41,42,43,44,45,46,47,48,49,50,51]. The majority of polymer flooding experiments conducted on polymer flooding EOR can be classified as core flooding experiments, polymer flooding in fractures, polymer application in microfluidics, or reservoir simulation of polymer flooding. It is important to note that polymers have been used in many other applications in the oil and gas industry, including in drilling fluids and fracture fluids, for conformance control, and even as a cement additive; however, the focus of this research is on polymer flooding enhanced oil recovery.

### 4.1. Core Flooding

Core flooding is a broad term that refers to the injection of fluid into a confined core plug. The plug can be cylindrical or rectangular depending on the core holder. The dimensions of the plug can vary significantly depending on the core holder capacity and the type of experiment being conducted. Core flooding can include continuous cores or cores with fractures. Fractures involve two distinct modes: either continuous or partial fracture. Continuous fractures involve a fracture that covers the entire length of the core plug. A partial fracture is a fracture that propagates partially across the core [34,35,36].

### 4.2. Microfluidics

Microfluidics studies the flow of the polymer through microchannels or porous media. The key difference between microfluidics and core flooding is the size of the observation, where microfluidics focuses on micro- and, sometimes, nanosized observations. Microfluidics is a key area of research with respect to the evaluation of the interactions occurring between polymers and rock, reservoir fluids, or both at the microscopic scale. Analysis of microchannels is usually conducted using imaging techniques such as magnifying microscopy, scanning electron microscopy, or transmission electron microscopy. There are several methods by which microfluidic channels can be created to model polymer flooding behavior in micropores. For all the methods summarized below, precision is key to producing a representative microfluidic rock sample for analysis [54,55,56,57,58,59,60,61,62,63].

- Etched Microchannels: Microchannels are etched on the surface of a slab of rock sample. Several etching methods can be used depending on the type of rock sample being studied and its properties. For example, acid etching can be used in limestone or dolomite rocks, whereas physical etching can be used in silicate cores such as sandstone.
- Crushed Microparticles: The use of crushed microparticles is considered one of the first methods by which microchannels were created. The rock sample is composed of microparticles that are crushed to a uniform size and non-uniform shapes. The crushed samples are cemented together using dissolved calcite or any other cementing material. Since the particles are non-uniform in shape and the rock particles are small microchannels are created between them. The size of the channels is a function of the size of the rock particles and their shapes. The smaller and more uniform the rock particles, the smaller the channel size.
- Lithological Coating: This method does not use rock as the main porous medium but instead relies on a fabricated microchannel medium that is then coated with a thin layer of rock. The fabricated microchannel can be modeled in any manner possible, which helps to control the size, length, and shape of the microchannel. The thin layer coating of the microchannel using rock ensures that any interactions between the rock and the fluid are incorporated in the setup during experimentation using the polymer or any other injected material or fluid.
- Bacterial Etching: Depending on the rock type, microorganisms can be used to create microchannels in the rock. This process can create heterogeneous microchannel networks in the rock surface; however, it is challenging to control the pathway of the microorganisms and also very difficult to limit their progression through the core. This method is more common in carbonate samples than in silicate samples.
- Precision Cutting: This is considered the most precise method in terms of modeling and creating a preplanned microchannel. Precision cutting is usually performed using plastic or acrylic media rather than a core plug. This method requires special machinery; however, it can be used to create replicas of any microchannel structure. This is extremely useful, especially in cases where a specific situation or reservoir characteristic is being studied. This can also be achieved on actual cores with specific characteristics that should be compatible with the precision etching device.

### 4.3. Reservoir Simulation

Reservoir simulation of polymer flooding projects involves studying the ability of the polymer to increase oil recovery at the entire field scale rather than the small core scale. The polymer should be defined in the reservoir simulation process based on its properties and characteristics. This can be achieved by creating a new phase with unique properties, the most important of which is viscosity. It is important to note that many reservoir simulators do not take into consideration polymer degradation based on the change in conditions, assuming an ideal polymer [34,35,36,37,38,39,40,41].

## 5. Polymer Flooding—Field Scale

At the field scale, many factors can impact polymer flooding. Some of these factors are common to the laboratory-scale tests; however, when analyzing field-scale polymer flooding, there are several unique characteristics that should be taken into consideration. These characteristics are mostly related to operational factors such as polymer injection and scale-based factors, which can impact the properties of the polymer during its propagation through the formation to displace the oil. Based on this, the field-scale polymer injection methods are discussed, along with some of the degradation mechanisms that can impact polymer performance during reservoir applications.

### 5.1. Injection Methods

The polymer flooding process begins with a preflush of fresh water into the reservoir, followed by a slug of 0.3 or higher pore volume of polymer solution, as shown in Figure 1, and another freshwater slug and continuous drive of low-salinity water injection. The injection of polymer solution between the two freshwater slugs is meant to minimize the direct contact between the polymer solution and reservoir saline water to avoid a reduction in polymer solution viscosity.

Characteristics of polymers as water mobility control agents have been reported in many studies [13,14,51,52,53,54,55,56,57]. Based on the principle and methodological description, polymer flooding does not significantly reduce the residual oil saturation. However, it improves oil recovery over water flooding by increasing the contacted reservoir volume. Also, in comparison to waterflooding, polymer flooding speeds up oil production and results in a higher recovery at breakthrough.

Polymer can be injected with other chemicals, such as alkaline and surfactants, to enhance the recovery. Alkaline–polymer flooding and alkaline–surfactant–polymer flooding have been discussed extensively by Khlaifat et al. [11]. When coupled with other chemical agents, the method by which the polymer is injected differs, including alteration of the order of injection based on the type of chemicals used. Table 1 provides a summary of the sequence of injection when polymer is coupled with another chemical compared to polymer flooding alone, as shown in Figure 1.

### 5.2. Polymer Hydrolysis

Hydrolysis is the process of breaking down bonds using water. It can be aggravated by many factors, including temperature, pressure, pH, salinity, and the type of energy input (such as electricity). After polymerization of the monomers, the polymer chains are susceptible to breakage due to external factors. When subjected to an elevated temperature, the polymer begins to hydrolyze. This results in the reduction in the length of the polymer chain; thus, the polymer begins to lose integrity and viscosity. Eventually, if excessive hydrolysis occurs, the viscosity of the polymer approaches that of water, nullifying the benefit of using a polymer compared to conventional water flooding. Hydrolysis should be taken into account when designing a polymer flooding operation through proper selection of the polymer based on the thermodynamic, rock, and fluid properties of the rock [13,14,15,16,17,18,19,20,21,22].

It is important to note that in some cases, partial hydrolysis of the polymer is preferred. When partial hydrolysis occurs in some polymers, it gains the polymer an effective functional group, which improves the overall performance of the polymer by reducing its degradation and increasing its ability to resist adsorption and dehydration. An example of this is HPAM. When injected, the polymer is partially hydrolyzed. The degree of hydrolysis usually does not exceed 40%. This allows the HPAM to gain a carboxylate group, which improves its overall performance. If the HPAM is hydrolyzed beyond 40%, it loses its integrity, and its viscosity decreases significantly [33,34,35,36,37,38,39].

### 5.3. Dehydration

Polymer dehydration is the process of the loss of water or fluid from the hydropolymer lattice. Loss of water can be due to several factors. An increase in temperature can result in significant and rapid polymer dehydration. The thermal stability of different polymers varies; therefore, dehydration should be tested to determine polymer compatibility with the reservoir. High pressure differentials can also result in polymer dehydration. This may be due to large depths, which require high injection pressure, or small pore sizes, which require high pressure for the polymer to propagate through the pores. Salinity is another significant factor that can impact polymer dehydration. When the formation water salinity is high compared to the hydropolymer water salinity, osmotic pressure causes the salt in the formation water to penetrate the polymer lattice. This results in polymer shrinkage and, therefore, loss of fluid. This can also result in polymer syneresis, which can damage the polymer structure and result in irreversible degradation [18,19,20,21,22,23,24,25,26,27].

### 5.4. Degradation

Polymer degradation involves permanent damage to the polymer chains. Degradation can occur due to many factors depending on the polymer type and properties. Polymer degradation involves the weakening or destruction of the polymer chains, resulting in the polymer losing its high viscosity, which is attributed to the long chains and the high molecular weight of the polymer prior to degradation. Degradation is a strong function of the polymer limitations and can occur due to excessive temperature, pressure differentials, pH, salinity (including both monovalent and divalent cations), gasses such as carbon dioxide or hydrogen sulfide, pore size distribution in the formation, or shearing of the polymer during injection [59,60,61,62,63].

### 5.5. Syneresis

Polymer syneresis is the process of the loss of fluid from the polymer, which results in an increase in polymer rigidity and a significant reduction in polymer mobility. Syneresis differs from dehydration in that syneresis is usually associated with polymer structural degradation. This results in difficulty for the polymer structure in reabsorbing the lost fluid; therefore, the polymer degrades beyond return. In contrast, if the polymer dehydrates, it can be rehydrated by the addition of fluid, usually water. Polymer syneresis is impacted by the same factors that impact polymer degradation and dehydration. It is the step that follows polymer dehydration. If excessive or repeated polymer dehydration occurs, syneresis eventually occurs. This can result in significant reductions in polymer workability, pumpability, injectivity, mobility, and overall sweep efficiency [52,53,54,55,56].

### 5.6. Shearing

During its injection, the polymer is subjected to extremely high shear rates. This shearing can damage the polymer chain and result in excessive polymer degradation. Some types of polymers can resist shearing more than others depending on their molecular structure. Vinyl acetate polymer can withstand some degree of shearing, for example, compared to weaker polymers such as acetic acid-based polymers. Although shearing cannot be entirely avoided, it can be reduced by proper design of the polymer injection on the surface. If the polymer is homogenously dissolved in the water, shearing tends to be significantly reduced. Decreasing the injection time and the injection pressure also tends to reduce polymer shearing [2,3,4,5,6,7,8,9,10].

### 5.7. Retention and Resistance Factor

**Figure 2:**
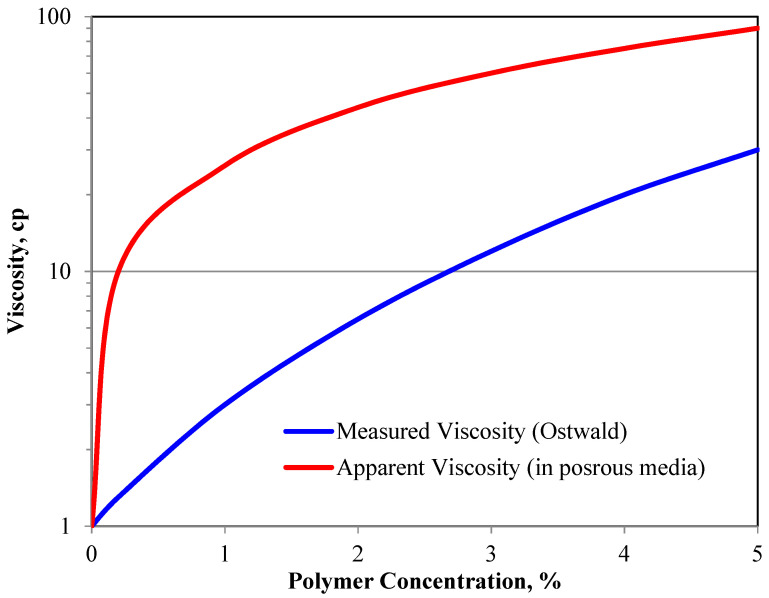
Measured polymer viscosity versus apparent viscosity while flowing in porous media.

As per field applications, the measured viscosity of low-concentration polymer solutions is only 1 to 1.5 cp. [35,53,54]. The viscosity of the polymer solution flowing in the reservoir is 5 to 25 times higher than the lab viscosity of the same polymer solution when calculated indirectly using Darcy’s equation (apparent viscosity), assuming the same effective permeability (Figure 2). It was observed that the behavior of low-concentration polymer solutions in porous media suggests a much higher apparent viscosity [13,64].

In reality, the effective permeability of the formation to water without a polymer is higher than its permeability to a polymer solution. However, it is difficult to distinguish between the effects of effective permeability reduction for a polymer solution and that of the apparent viscosity increase. Reservoir engineers are usually concerned with the total effect that results in mobility reduction as measured by the resistance factor (R) and calculated as follows:

$$R=\frac{\lambda_{w}}{\lambda_{p}}=\frac{k_{rw}/\mu_{w}}{k_{rp}/\mu_{p}}=\frac{k_{rw}\mu_{p}}{{\mu_{w}k}_{rp}}=\frac{M_{w-o}}{M_{p-o}}$$

where λ_p_ is water-soluble polymer mobility; k_rw_ and k_rp_ are permeabilities relative to water and polymer solution, respectively; μ_p_ is the viscosity of the polymer solution (apparent viscosity); and M_w−o_ and M_p−o_ are the water–oil and polymer solution–oil mobility ratios, respectively.

**Figure 3:**
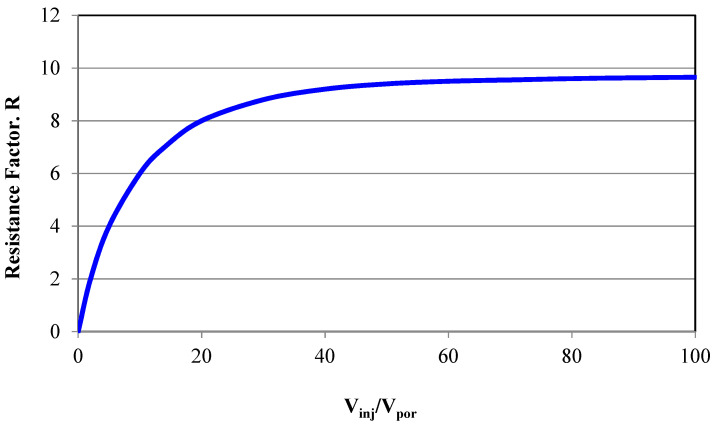
Resistance factor (R) as a function of cumulative injected volume.

The resistance factor (R) is usually plotted as function of the ratio of the cumulative injected volume per porous medium volume (V_inj_/V_por_), as shown in Figure 3.

It was experimentally observed by Pye [55] injecting a 300 ppm polymer solution through a porous core plug resulted in a rapid increase in the resistance factor from 1 to 8 for the first 20 pore volumes injected. Subsequent injection of polymer solution did not affect the resistance factor. The tendency of the resistance factor to stabilize (Figure 3) must be tested in a lab for each reservoir rock and fluid to avoid high injection pressures or blockages during field application.

Injecting polymer solution with high resistance factors is beneficial in the case of plugging the more permeable streak zones near injectors and to reduce the variation in permeability. Plugging high-permeability reservoir zones far from the injectors can be achieved via cross linkers in the gel polymer solution.

After polymer injection, the reduction in the permeability of the rock to water can be estimated according to the residual resistance factor (R_R_), which is calculated as:

$$R_{R}=\frac{\left(\frac{k_{rw}}{\mu_{w}}\right)\;before\;polymer\;flow}{\left(\frac{k_{rw}}{\mu_{w}}\right)\;after\;polymer\;flow}$$

Usually, permeability reduction is observed after flushing with low-salinity water (brine), as seen in Figure 1, following the injection of a polymer solution into the reservoir. The reduction in reservoir permeability occurs due to polymer adsorption by the rock surface and through mechanical entrapment of polymer molecules. Unfortunately, this process is irreversible, which could be classified as a form of formation damage.

The residual resistance effect continues after the polymer flooding process is over; hence, it has a cost-effective significance, as no extra money is expended on polymer injection. Not all polymers exhibit a residual resistance effect; for example, biopolymer polysaccharides are not adsorbed on rock surfaces, so when injected, their residual resistance factor is negligible.

A comprehensive review of polymer retention mechanisms and relevant factors, as well as modeling techniques and methods applied to estimate polymer retention, was carried out by Al-Hajri et al. [65].

**Figure 4:**
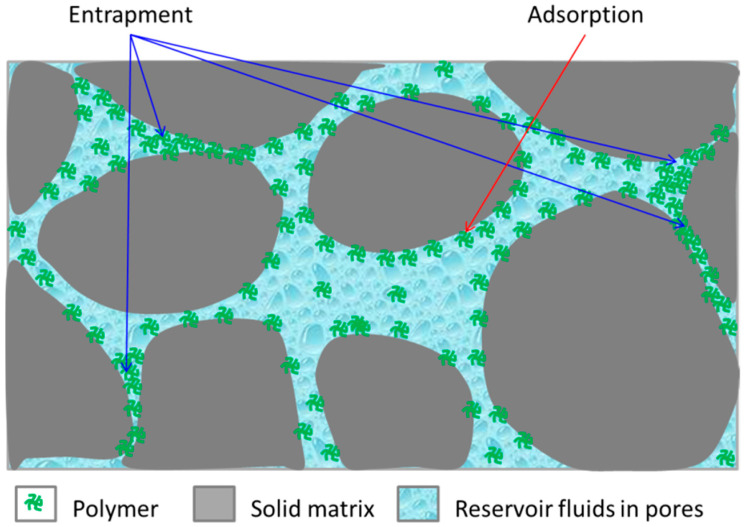
Polymer retention mechanisms (adsorption and entrapment).

As mentioned with respect to permeability reduction above, a polymer can be retained in the reservoir by two mechanisms, as shown in Figure 4, namely (1) by adsorption of the polyacrylamide on rock surfaces and (2) by entrapment/deposition of polymer molecules in small pore spaces. The polyacrylamide polymer is adsorbed on the surface of most rock reservoirs [66]. Some rocks, such as calcium carbonate, have a greater affinity for polymers than silica. This can result in a significant loss of the provided kinetic energy to the polymer, which, in turn, can impact the performance of the polymer flooding operation.

The polymer layers absorbed on the solid grains, as shown in Figure 4, represent both an additional resistance to flow and the loss of polymer. Polymer adsorption results in a reduction in the concentration of polymer solution that flows back to the surface. The lower the polymer concentration before flowing through the porous space, the lower the adsorption on the rock surface; the opposite is also true.

The porous space in the reservoir rock, as shown in Figure 4, offers a variety of pore sizes (openings) and pore size distribution. The long chain of the polymer molecule can easily flow through a large pore space but cannot leave it if the other end has a smaller opening (size). Then, the polymer molecule becomes trapped. Also, when the reservoir fluid flow is restricted or stopped, entrapment occurs. During entrapment, the polymer molecule loses its elongated shape and coils up, allowing only the flow of brine through the porous space.

In a reservoir formation, there are small openings (tight pore space) that are not contacted by flowing polymer molecules, forming the so-called inaccessible pore volume [58]. Almost 30% of the total pore volume of the porous space may not be accessible to polymer molecules [58,59].

This inaccessibility allows polymer solutions to advance farther and displace oil at a faster rate than predicted. In other words, the effective porosity for brine is greater than the effective porosity for polymer solution.

### 5.8. Environmental Concerns

Synthetic polymers can be harmful to the environment [4]. Therefore, the use of polymers to enhance oil recovery requires in-depth environmental impact assessment. Polymer degradation limits the use of such chemicals under harsh reservoir conditions such as high temperature and high pressure, in which cases polymers are not environmentally friendly and uneconomical [67]. One of the potential environmental impacts associated with enhanced oil recovery using chemical methods is the contamination of ground water and the pollution of surface waters from leaks and/or spills of oil and brine that contain chemicals [68]. Environmental planning and impact assessment must be integrated into project development to ensure an environmentally acceptable polymer flooding project with the aim of enhancing oil recovery.

### 5.9. Factors Impacting Field Applications of Polymer Flooding

As per fluid dynamics, the capillary number (C_a_ = μV⁄σ) is a dimensionless quantity representing the relative effect of viscous drag forces (μV, where μ is the dynamic viscosity of the liquid and V is the characteristic velocity) versus surface tension forces (σ) acting across an interface between a liquid and a gas or between two immiscible liquids. Therefore, an increase in the viscosity of the injected liquid (as compared to just water) results in an increase in the capillary number in the porous media. In other words, the capillary forces become negligible compared to viscous forces [65,69]. A water-soluble polymer added to water at a concentration of 0.01–0.1% results in an increase in the viscosity of polymer solution of three to four times [70]. Consequently, the apparent viscosity of the polymer while flowing through the pores increases by up to 20 times. This increase in viscosity makes polymer solutions effective for utilization in fields involving the use of high-viscosity and heterogeneous reservoirs.

Water-soluble polymers used in polymer flooding are, by themselves, anionic surfactants [71,72]. The propagation of surfactants in the reservoir reduces the surface tension [71,72,73,74,75,76]. If the polymer solution contains more surfactant additives, the resulting complex of polymer molecules and surfactants has a molecular weight greater than the molecular weight of the polymer and, therefore, a higher-viscosity polymer solution. The decrease in interfacial surface tension causes the residual oil to wash off of the surface of the reservoir rock pores behind the displacement front [77,78].

The paramount influence on the efficiency of polymer flooding is produced by the non-Newtonian characteristic of the polymer solution [79,80,81,82,83]. Water-soluble polymers commonly used in oilfield practice are characterized by shear thinning or pseudoplastic rheology [83], which results in low viscosity at high speeds of flow through porous media and maximum viscosity for very slow flows of liquid [83].

The most significant component of the EOR application cost is the polymer itself. Although conventional polymers such as HPAM are not costly, the need to synthesize a complex co- or terpolymer using high-cost chemicals can increase the overall polymer flooding cost. The loss of polymer in the formation due to adsorption is also a major addition to the cost. Finally, polymer recycling is extremely difficult due to the excessive degradation of the polymer structure, which makes it incompatible with the newly injected polymer. Some studies have investigated the usage of recycled polymer as sacrificial polymer through which the recycled polymer is adsorbed by the formation instead of the newly injected polymer. Other costs of the operation include mixing tanks; pumps for injection; and hazardous waste processing, storage, and transportation. Polymer flooding can increase the recovery factor (RF) over waterflooding by up to 20% of the original oil in-place (OOIP), at only USD 3 to 6 per extra barrel of oil [30].

## 6. Discussion

The application of polymer flooding depends on the properties of both reservoir rocks and reservoir fluids. This means that the polymer flooding process must be properly evaluated before it can be applied to any reservoir. The evaluation process is needed for feasibility purposes and may consist of laboratory experiments, modeling and simulation, economic analysis, and pilot testing.

The influence of reservoir characteristics on polymer flooding is discussed extensively in [55,84]. The reservoir depth is a critical factor only with respect to reservoir temperature. The behavior of the polymer solution is stable if the reservoir temperature is less than 200 °F [85]. If the injection pressure of the polymer solution is less than the formation parting pressure and is not so high that it requires expensive pumping equipment, then the reservoir pressure is not critical [55]. The porosity of the reservoir rock must be medium to high (higher than 18%) to assure a good storage capacity [84]. The acceptable range of the absolute permeability of the reservoir rock is between 50 and 250 md [64]. Low absolute permeability (less than 50 md) requires higher injection pressures. Permeability values greater than 250 md and less than 1000 md are considered ideal. As the permeability increases beyond 1000 md, conventional water flooding results in high recoveries that make polymer flooding expenses unjustifiable [64]. To determine applicable areas for polymer flooding, permeability variation is used instead of absolute permeability. It was determined that heterogeneous reservoirs are the best candidates for polymer flooding for several reasons, such as [86,87] the reduction in the rock’s permeability caused by polymer solutions and the tendency of the solution to flow in the direction of unswept reservoir areas.

Guidelines developed by Chang [88] and Taber et al. [88,89,90] indicate the suitability of polymer flooding for viscous oils up to 120 to 200 cp. When the oil viscosities of reservoirs are high, thermal methods become competitive. Higher mobile oil saturation at the start of the project is achieved by close-to-zero water–oil ratios. This means that polymer flooding is more successful when applied from the very beginning as a secondary recovery technique rather than water flooding. Polymer solution flooding is efficient for oil with a gravity range of 13 to 42.5 °API, with an average value of 26.5 °API [91].

Usually, any enhanced oil recovery process selection criteria serve as the first-pass screening guidelines to compare the candidate reservoir with other reservoirs produced using the same EOR method. The screening criteria for polymer flooding are shown in Table 2 [92,93,94,95,96,97]. The maximum polymer flooding viscosity (4000 cp) was reported in Pelican Lake, Canada [97].

Several polymer flooding projects and experiments have been implemented worldwide in order to increase oil recovery and reduce water production through mobility control [86,98,99,100,101,102,103,104,105,106,107,108,109,110,111,112,113,114,115,116,117,118,119,120]. A summary of some field studies conducted in different countries worldwide using polymer flooding is provided in Table 3. It is important to note that although some field tests have been conducted using biopolymers—mainly xanthan gum—the majority of field applications using polymers rely on hydrolyzed polyacrylamide as the polymer agent. Table 4 provides a summary of polymer flooding applications in some other fields [121,122,123,124,125,126,127,128,129,130,131,132,133,134,135].

## 7. Conclusions

We performed a review of the main factors impacting polymer flooding applications in field studies and laboratory-scale applications. The main findings of this research are summarized as follows.

1. Polymer-enhanced oil recovery is one of the most applied EOR methods in terms of both laboratory studies and field applications. The main factors impacting polymer injection are tied to polymer selection, reservoir rock and fluid properties, polymer concentration, and injection methodology.
2. Polymer degradation is represented in multiple steps depending on the severity of impact. This can include the less severe dehydration and partial hydrolysis and the more severe shearing and syneresis.
3. When coupled with other chemical EOR methods, polymers can be used in two main manners: either as a mechanism to support oil recovery by increasing sweep efficiency or as a sacrificial polymer to avoid the loss of the more costly surfactant to the formation.
4. Some polymers can pose severe environmental concerns, especially if they react with underground fluids or break down into hazardous chemicals. It is therefore a major recommendation of this research that proper chemical analysis and interaction analysis be conducted as part of the polymer selection process.

## Figures and Tables

**Table 1:** Polymer injection methods based on application.

Injection	Sequence	Main Mechanism
Polymer–Alkaline	Polymer is injected prior to the alkaline agent	The polymer is injected to mobilize the crude oil and improve the areal and vertical sweep efficiencies. The alkaline agent is then injected to enhance the displacement efficiency.
Polymer–Surfactant	Polymer is injected prior to the surfactant	The polymer is injected to mobilize the crude oil and improve the areal and vertical sweep efficiencies. The polymer is sometimes adsorbed on the surface of the rock. This reduces the loss of surfactant to the rock through adsorption. The polymer in this case is referred to as a sacrificial polymer, since a volume of it is sacrificed to avoid excessive loss of the costly surfactant.
Polymer–Alkaline–Surfactant	The sequence depends on the application and rock and fluid properties	This method is applied when there is benefit from all three chemical methods. The polymer improves the areal and vertical sweep efficiencies. The alkaline and surfactant improve the displacement efficiency. The alkaline agent is used if the oil is acidic in order to generate an in situ surfactant. This can reduce the requirement for surfactant flooding, improving the overall economics of the project.
Polymer–Low-Salinity Water	The low-salinity chase water is injected after the polymer	Low-salinity water flooding has been shown to improve areal and vertical efficiency, along with slight improvement in the displacement efficiency. When combined with polymer flooding, it can help prolong the life of the polymer by reducing polymer degradation due to salinity. Low-salinity chase flooding can also slightly improve displacement efficiency.

**Table 2:** EOR screening criteria for polymer flooding [92,93,94,95,96,97].

Oil Properties		Reservoir Characteristics					
Viscosity, cp	Gravity, °API	Porosity, %	Oil Saturation (% PV)	Formation Type	Permeability, md	Depth, ft	Temperature, °F
0.4–4000	13–42.5	10.4–33	34–82	Sandstone	1.8–5500	700–9460	74–237.2

**Table 3:** Summary of prominent polymer field applications.

Field	Lithology	Temp, C	Oil Viscosity, cp	Polymer	Polymer Conc., ppm	Brine TDS, ppm	Reference
Xiaermen Field, China	Unconsolidated Sand	50	70	HPAM	1400	2127	[98]
Daqing Field, China	Fluvial Sandstone	45	-	HPAM	2000	-	[99]
East Bodo Reservoir, Canada	Sandstone	23	600–2000	HPAM	1500	25,000–29,000	[100]
Tambaredjo Field, Suriname	Sandstone	36	1260–3057	HPAM	1000	400–500	[101]
Marmal Field, Oman	Sandstone	46	40–120	HPAM	1000	600	[102]
Vacuum Field, USA	Dolomite	38	0.88	HPAM	50	-	[103]

**Table 4:** Polymer flooding field applications.

Region	Country	Field	EOR Class	EOR Type	Operator
AsiaPac	China	Fuju	Chemical	Polymer	CNPC
AsiaPac	China	Dagang	Chemical	Polymer	CNPC
AsiaPac	China	Xinjiang	Chemical	Polymer, Surfactant	CNPC
AsiaPac	China	Shengli	Chemical	Polymer, Surfactant	Sinopec
AsiaPac	China	Henan	Chemical	Polymer, Surfactant	Sinopec
AsiaPac	China	BoHai/Yanchang	Chemical	Polymer	CNOOC
AsiaPac	India	Mangala	Chemical	Polymer	Cairn
CSAM	Colombia	Chichimene	Chemical	Polymer	Ecopetrol
CSAM	Colombia	Palogrande	Chemical	Polymer	Ecopetrol
Eurasia	Russia	West Salym, Upper Salym, Vadelyp	Chemical	Polymer	Salym Petroleum NV
Eurasia	Russia	Romashkinskoye	Chemical	Polymer	Tatneft
Europe	UK	Schiehallion (Quad 204 projects)	Chemical	Polymer	BP
Middle East	Oman	Marmul	Chemical	Polymer	PDO
North America	Canada	Pelican Lake	Chemical	Polymer	CNRL
North America	Canada	Pelican Lake	Chemical	Polymer	Whitecap
North America	United States	North Burbank	Chemical	Polymer	unknown
